# Preoperative Activation of c-Src Kinase in Atrial Tissue in Patients Developing Postoperative Atrial Fibrillation

**DOI:** 10.3390/medicina61091669

**Published:** 2025-09-15

**Authors:** Tomasz Andrzej Bonda, Magdalena Dziemidowicz, Tomasz Hirnle, Iwona Dmitruk, Izabela Bialuk, Maria Małgorzata Winnicka

**Affiliations:** 1 Department of General and Experimental Pathology, Medical University of Bialystok, ul. Mickiewicza 2c, 15-222 Białystok, Poland; 2 Department of Cardiosurgery, Medical University of Bialystok, ul. Skłodowskiej-Curie 24a, 15-276 Białystok, Poland

**Keywords:** atrial fibrillation, postoperative arrhythmia, intracellular signaling, cardiomyocyte, pathophysiology

## Abstract

*Background and Objectives*: Atrial fibrillation (AF) is a common complication of cardiac surgery. c-Src has been implicated in atrial remodeling in chronic AF, but its role in the early postoperative setting remains unclear. We, therefore, investigated whether baseline c-Src expression in atrial tissue is associated with the subsequent development of postoperative AF (PoAF). The aim of the present work was the evaluation of atrial c-Src expression and activity in patients subjected to open heart surgery who were previously free from AF and to check if changes to the initial level of this protein predispose to the development of postoperative AF (PoAF). *Materials and Methods*: Forty-two patients without previous AF history we enrolled. Patients with an AF episode during postoperative in-hospital follow-up were assigned to the PoAF group, while the rest (in sinus rhythm—SR) constituted the control group. Samples of the right atrial appendage were harvested before the introduction of the extracorporeal circulation. The expression of c-Src and phospho-c-Src(Tyr416), as well as upstream regulators of c-Src kinase, STAT3, ERK1/2, PDGFRα, and PDGFRβ, was assessed using Western blot. *Results:* AF occurred in 14 subjects. Expression of c-Src and phospho-c-Src was significantly higher in the PoAF group than in the SR group (c-Src: 1.65×, *p* = 0.037, and phospho-c-Src: 2.75×, *p* = 0.003). In addition, in the right atrium of PoAF patients, there was significantly elevated expression of STAT3, ERK1/2, and PDGF receptors, which may facilitate activation of c-Src kinase in patients with PoAF. *Conclusions*: Our preliminary findings suggest that c-Src expression and activity may contribute to atrial vulnerability and could represent a molecular target for future therapeutic interventions to prevent PoAF.

## 1. Introduction

Atrial fibrillation (AF) is the most common sustained arrhythmia in clinical practice and the most frequent arrhythmic complication after cardiac surgery. It affects up to 30% of all operated patients, being less common after coronary artery bypass grafting (CABG) alone (about 22%) but about twice as frequent in patients undergoing combined CABG and valve surgery [1,2]. Postoperative AF (PoAF) compromises cardiac performance and may cause hemodynamic instability, prolong ventilatory support and hospitalization, and reduce both early and late survival [1,3]. Preoperative risk factors for AF include older age, hypertension, prior heart failure, mitral valve disease, and increased atrial diameter. Procedure-related risk factors include the extent of surgery and increased levels of inflammatory and oxidative stress markers [4,5]. AF can be initiated by premature atrial ectopic beats resulting from enhanced automaticity or triggered activity [6,7]. Its perpetuation is favored by electrical and structural remodeling of the atria, which slows conduction and shortens the action potential [6,8,9]. Electrical remodeling involves downregulation of the slow inward calcium current (I_CaL_) and upregulation of outward potassium currents [10]. Phosphorylation of ion channel proteins by kinases such as protein kinase A, protein kinase C, and Src family kinases is a key regulatory mechanism. Src family kinases can phosphorylate L-type calcium channels and potassium channels carrying ultrarapid, rapid, and transient outward currents [11]. Previous studies have demonstrated that chronic AF is associated with increased expression and phosphorylation of c-Src [12,13]. In addition, inhibition of c-terminal Src kinase (which normally suppresses Src activity) by ibrutinib stimulates atrial remodeling and increases AF risk [14]. Members of the Src kinase family act as intracellular signaling hubs with pleiotropic effects through pathways such as extracellular signal-regulated kinases (ERKs) or phosphatidylinositol 3-kinase/protein kinase B (PI3K/Akt). Previous studies have implicated c-Src activation in chronic AF, but its role in the early postoperative setting without pre-existing AF remains unclear. As many of these activities dynamically alter the electrophysiologic background, we hypothesized that elevated c-Src activity during the periprocedural period may contribute to the development of PoAF.

The aim of this study was to prospectively assess c-Src expression and activation in atrial tissue of patients without prior AF undergoing cardiac surgery and to examine its association with the occurrence of PoAF.

## 2. Materials and Methods

Consecutive patients in a stable hemodynamic state subjected to elective open-heart surgery were screened before the operation, and only patients with a normal sinus rhythm and without a previous history of any AF episode were included. Patients with ongoing inflammatory processes/increased levels of C-reactive protein or unstable coronary syndromes were excluded. Warm blood cardioplegia was used in all patients. During the postoperative period, patients were monitored for episodes of AF. During the first 48 h after the surgery, the ECG was monitored constantly; the later evaluation was based on clinical assessment and at least a daily 12-lead ECG strip to confirm either sinus rhythm or AF. Subjects with AF episodes lasting for at least 30 min during the postoperative in-hospital follow-up were assigned to the PoAF group, and those with a normal sinus rhythm throughout the in-hospital stay constituted the control group (SR group). The minimal period of screening for AF was 6 days following the surgery.

Samples of the right atrial appendage were harvested during the initial period of operation before the introduction of extracorporeal circulation. Tissue samples were flushed with ice-cold saline and snap-frozen in liquid nitrogen and processed as previously described [15]. Briefly, samples were defrosted on ice and homogenized in the ice-cold RIPA buffer (Sigma, St. Louis, MO, USA, #R0278) with the addition of phosphatase inhibitors (sodium orthovanadate and sodium fluoride) and protease inhibitors (Sigma, St. Louis, MO, USA, #P8340). Total protein concentrations were measured by the spectrophotometric Bradford method. A total of 50 μg of protein was loaded per lane on the polyacrylamide gel, and following electrophoretic separation, proteins were transferred onto the nitrocellulose membrane. Membranes were blocked using 5% non-fat milk in phosphate-buffered saline and probed with specific primary antibodies (Abs), washed, and incubated with appropriate secondary Ab conjugated with horseradish peroxidase. In the next step, membranes were incubated with the chemiluminescent substrate (SuperSignal West Pico PLUS Chemiluminescent Substrate, Thermo Fisher Scientific, Waltham, MA, USA) and exposed to X-ray film. Details of the above procedures are described in our previous work [15]. Levels of c-Src protein and phospho-c-Src (with activating phosphorylation at Tyrosine 416), as well as upstream regulators of c-Src kinase, Stat3, ERK1/2, and PDGFRα and PDGFRβ (platelet-derived growth factor receptors α and β), were assessed. The primary Abs used were from SantaCruz Biotechnology (Dallas, TX, USA): anti-c-Src (sc-18), anti-phospho-c-Src (sc-81521), anti-ERK1/2 (sc-292838), anti phospho-ERK1/2 (sc-101761), anti-PDGFRα (sc-338), anti-phospho-PDGFRα (Tyr720; sc-12910), anti-STAT3 (sc-7179), anti-phospho-Stat3 (Ser727, sc-71792), anti-PDGFRβ (sc-432), anti-phospho-PDGFRβ (Tyr740; sc-17173). Levels of the above-mentioned proteins were normalized to the housekeeping protein β-actin (Cell Signaling Technology, Danvers, MA, USA, #4970) to account for loading variability. Next, the mean normalized expression value of the SR group was calculated, and finally, all individual normalized values were divided by the SR group mean, resulting in relative expression values where the SR group average equals 1. This three-step normalization allowed for direct comparison of protein expression between groups while controlling for inter-sample variability. Quantitative analysis of blots was performed with ImageJ Software v.1.46r. Expression of proteins in the PoAF group was related to the level of proteins in the SR group, in which the expression was set as 1.

All procedures performed in studies involving human participants were in accordance with the ethical standards of the institutional ethical committee (Bioethics Committee of the Medical University of Bialystok, R-I-002/84/2008, approval date 28 February 2008 ) and with the 1964 Helsinki declaration and its later amendments or comparable ethical standards. Informed consent was obtained from all individual participants included in the study.

### Statistical Analysis

The study has an exploratory design without an a priori power analysis due to unavailable effect size estimates. The results were presented as the mean ± standard deviation (SD) unless otherwise stated. Sample distribution was tested using the Lilliefors test. As data was not normally distributed, the Mann–Whitney test was used for statistical analysis, and *p* < 0.05 was considered statistically significant. Correlations were estimated using Spearman’s test, and for this method, *p* < 0.01 was considered significant. A binary logistic regression model was applied to estimate the independent effects of selected variables on the likelihood of PoAF development. All analyses were performed using Statistica 13 (StatSoft Inc., Tulsa, OK, USA).

## 3. Results

AF occurred in 14 patients (PoAF group, 33% of all analyzed), and the remaining 28 patients maintained sinus rhythm (SR group). The groups did not differ in basic demographic and echocardiographic parameters, with the exception of higher left atrial diameter and more frequent treatment with spironolactone in patients from the PoAF group. Detailed characteristics of the groups are shown in Table 1.

**Figure 1:**
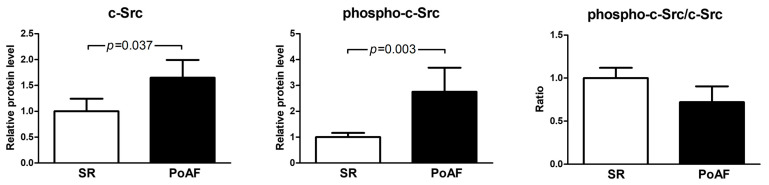
Relative expression of c-Src protein (left graph) and relative level of phosphorylated c-Src (middle graph) were higher in the PoAF group. The ratio of phosphorylated-to-total protein is shown on the right graph. Protein levels were normalized to β-actin and then scaled relative to the mean value of the SR group, which was set to 1. Statistical comparisons were performed using the Mann–Whitney U test. Bars represent mean values ± SEM.

Expression of c-Src and phospho-c-Src was significantly higher in the PoAF group than in controls. c-Src relative expression in PoAF patients was 1.65 ± 1.28 as compared to 1.0 ± 1.28 in the SR group (*p* = 0.037). The level of phosphorylated c-Src was also higher in the PoAF group (2.75 ± 3.5 vs. 1 ± 0.87 in the SR group, *p* = 0.003). The relative abundance of c-Src, phospho-c-Src, and their ratio is presented in Figure 1.

In the univariate analysis (Mann–Whitney test), two clinical variables, along with c-Src and phospho-Src, differed significantly between the PoAF and SR groups. To further assess their independent contributions, we performed a binary logistic regression including these four factors. In this model, spironolactone treatment remained independently and positively associated with PoAF occurrence. However, due to the very low absolute number of patients receiving this treatment, we consider this finding an error rather than a valid outcome of the model. The remaining variables showed no significant associations with PoAF in this model (Wald statistic range: 0.8–1.8; all *p*-values > 0.1).

**Figure 2:**
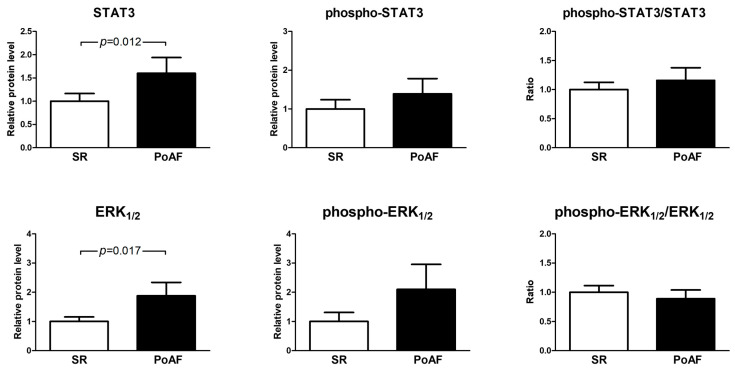
Relative expression of STAT3 protein and ERK1/2 kinases was higher in the PoAF group (upper and lower left graphs, respectively), while their phosphorylation was comparable between groups (right graphs). Protein levels were normalized to β-actin and then scaled relative to the mean value of the SR group, which was set to 1. Statistical comparisons were performed using the Mann–Whitney U test. Bars represent mean values ± SEM.

Expression of c-Src downstream signaling mediators STAT3 and ERK1/2 was significantly higher in the PoAF group (1.60 ± 1.27 vs. 1.0 ± 0.86; *p* = 0.012, and 1.87 ± 1.72 vs. 1.0 ± 0.82; *p* = 0.017, respectively). The levels of phosphorylated (active) forms of both STAT3 and ERK1/2, although showing an upward trend in the PoAF patients, were not significantly different between groups (Figure 2).

**Figure 3:**
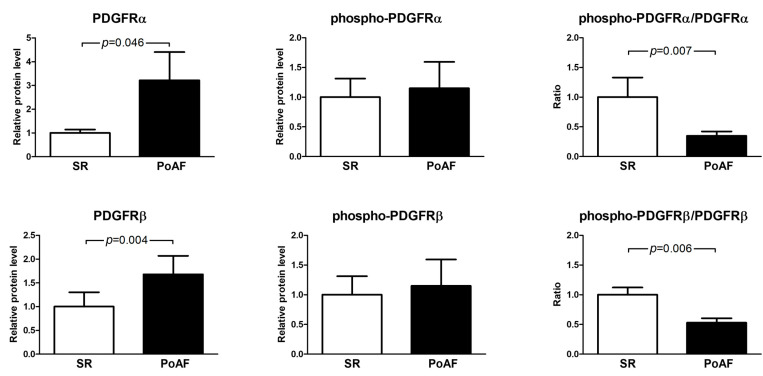
The expression of PDGF receptor subtype alpha (upper left) and beta (lower left) was higher in the PoAF group. The fraction of the phosphorylated form of both these receptors was diminished in the PoAF group (upper right and lower right graphs, respectively). Protein levels were normalized to β-actin and then scaled relative to the mean value of the SR group, which was set to 1. Statistical comparisons were performed using the Mann–Whitney U test. Bars represent mean values ± SEM.

Expression of important upstream Src regulators, such as PDGF (platelet-derived growth factor) receptor subtypes α and β, was also higher in PoAF patients (PDGFRα: 3.2 ± 4.42 vs. 1.0 ± 0.76; *p* = 0.047; PDGFRβ: 1.68 ± 1.46 vs. 1.0 ± 0.63; *p* = 0.004). The amounts of phosphorylated PDGF receptors in the PoAF group were not significantly different than those in the SR group. In the PoAF group, we observed a significantly lower ratio between phosphorylated and total abundances of these receptors (phospho-PDGFRα/PDGFRα ratio in PoAF group was 0.35 ± 0.27 vs. 1.0 ± 1.7 in SR group, *p* = 0.007; phospho-PDGFRβ/PDGFRβ: 0.53 ± 0.28 vs. 1.0 ± 0.65 in the PoAF and SR groups, respectively, *p* = 0.006; Figure 3). Low percentage of phosphorylated PDGFRs suggests compensatory silencing of the PDGF pathway; however, elevated expression of the receptors may constitute a foundation for fast induction and overactivation of this pathway during or after the operation.

Correlation analysis was performed to evaluate the strength of associations between c-Src and its upstream (PDGFRα/β) and downstream (STAT3, ERK1/2) regulators, aiming to better understand coordinated molecular signaling in atrial tissue. In the PoAF group, total c-Src levels showed strong positive correlations with all examined proteins, indicating synchronized activation of the Src pathway. Notably, the strongest associations were observed with ERK1/2 and PDGFRα, suggesting their key role in initiating the signaling cascade. Phosphorylated c-Src correlated primarily with phosphorylated STAT3 and PDGFRα, reflecting selective activation of these components in patients who developed PoAF. In contrast, the SR group exhibited weaker and less consistent correlations. No significant association was found between c-Src and ERK1/2, and phosphorylated c-Src correlated only with phosphorylated STAT3 and PDGFRβ. In patients who developed PoAF, the correlation profile suggests a tightly coordinated signaling network involving c-Src and its regulators, potentially reflecting a preoperative molecular predisposition to arrhythmogenesis. In the SR group, the signaling appears more fragmented and less integrated, consistent with a physiologically quiescent atrial state. Detailed Spearman correlation coefficient values are presented in Table 2.

## 4. Discussion

Atrial fibrillation is a common complication after cardiac surgery. Multiple clinical variables were identified as the prognostic factors predisposing to this arrhythmia [16]. PoAF is provoked by the interplay between different variables, which are patient- and/or procedure-dependent and translate into metabolic, inflammatory, hemodynamic, and mechanical factors. The predisposition for initiation and perpetuation of FA depends largely on the internal properties of the atrial tissue, and these are regulated by the intracellular signaling cascades. Electrophysiological properties of the atrial tissue can change dynamically due to metabolic derangements, like electrolyte imbalances, pH changes, or ATP depletion, but also because of posttranslational modifications of the protein building pumps or ionic channels, which affect the kinetics of ion transfer. Reversible phosphorylation of proteins forming ionic channels is the crucial mechanism of their regulation. The role of tyrosine kinase in the modulation of the electrophysiological substrate of arrhythmia has received attention in recent years.

Src family protein kinases belong to the non-receptor tyrosine kinase family and consist of c-Src, Fyn, Yes, Yrk, Blk, Fgr, Hck, Lck, and Lyn proteins. They are activated in response to stimulation of a variety of cell surface receptors, including receptors possessing tyrosine kinase activity, integrin receptors, and G protein-coupled receptors. Activation of c-Src kinase requires its phosphorylation at tyrosine residue in position 416, which is located in the catalytic domain of the protein [17].

Our principal finding is the preoperative upregulation and activation of c-Src kinase in the right atrial tissue of patients who subsequently developed PoAF. The magnitude and consistency of the molecular signal—elevated total c-Src, increased phospho-c-Src(Tyr416), and concordant rises in STAT3, ERK1/2, and PDGFRα/β with strong inter-protein correlations—argue for a biologically meaningful alteration of atrial signaling prior to the arrhythmic event. To our knowledge, this is the first study to demonstrate preoperative activation of Src signaling in atrial tissue of patients who subsequently develop PoAF. While a multivariate logistic model of the baseline clinical variables identifies neither c-Src nor phospho-c-Src as the independent factor related to PoAF, this does not exclude the mechanistic relevance of the Src pathway. Our results suggest that atrial tissue from PoAF patients exhibits a robust molecular phenotype consistent with Src pathway activation, providing a plausible mechanistic substrate linking preoperative myocardial state to postoperative arrhythmogenesis.

The range of c-Src kinase substrates is very wide and includes proteins crucial for the regulation of cell survival, proliferation, and angiogenesis or motility, as well as proteins regulating ion transfer through the plasma membrane and ionic fluxes between the intracellular compartments. Experiments in mice reveal the role of Src in angiotensin II signaling by potentiating its hypertensive response, promoting oxidative stress, endothelial, and left ventricular dysfunctions [18]. Cardiac surgery with cardiopulmonary bypass circulation (CPB) has no significant influence on c-Src expression or activity [19]; thus, it seems to be a relatively procedure-independent risk factor for postoperative AF. However, MEK/ERK signaling, which is downstream of Src, was shown to be downregulated after CPB, probably as a result of increased expression of mitogen-activated protein kinase phosphatase 1 (MKP-1) [19].

Based on previous publications, several potential mechanisms linking c-Src kinase with atrial fibrillation may be considered. First, c-Src is involved in the modulation of the sympathetic inputs, which play an important role in the initiation and perpetuation of atrial arrhythmias, including atrial fibrillation [20]. Increased sympathetic tone is the most important arrhythmogenic factor after cardiac surgery, and inhibiting its influence on the heart with short-acting β-blockers is very effective in terminating AF following CABG [21,22]. Adrenergic stimulation activates the cAMP-dependent signaling cascade and simultaneously activates Src kinases [23]. Enhanced automaticity of the sinoatrial node induced by sympathetic stimulation is facilitated by Src, and a blockade of Src tyrosine kinases with a selective inhibitor suppresses the automaticity of these cells [24]. On the other hand, the inhibition of tyrosine kinases with an unselective inhibitor was shown to decrease conductance of potassium channels involved in the regulation of the resting membrane potential [25]. Another link between adrenergic stimulation and Src-dependent mechanisms in the pathogenesis of AF may be the altered regulation of the cellular calcium turnover. AF is characterized by diminished transmembrane calcium fluxes and defective calcium release from the sarcoplasmic reticulum [26]. The L-type calcium channel is the most important carrier of extracellular calcium. A reduction in the calcium current through this channel (I_Ca,L_) shortens the action potential duration and plays an important role in both the development, and, more importantly, the perpetuation of AF [27]. In chronic AF I_Ca,L_ is significantly diminished [28], and in patients without a history of AF, the postoperative arrhythmia was predicted by lower I_Ca,L_ density in atrial cells [28]. Some well-established conditions predisposing to AF, like mitral valve disease or reduced left ventricular ejection fraction, are characterized by downregulation of I_Ca,L_, which is, however, more dynamically activated in response to sympathetic stimulation, suggesting the role of transient cellular calcium overload for the development of AF [28,29]. Src kinases inhibit PKA-related activation of I_Ca,L_, which may be considered a mechanism protecting from calcium overload under excessive sympathetic stimulation, but it is typically seen in AF [23]. Src also mediates the inhibition of I_Ca,L_, which is induced by the macrophage migration inhibitory factor (MIF). This cytokine is released by atrial myocytes under oxidative stress, diminishes the I_Ca,L_ amplitude, and decreases the expression of the pore-forming α1C subunit of the L-type Ca^2+^ channel [13,30]. During oxidative stress, activated Src, in addition to protein kinase C, upregulates the synthesis of MIF by atrial myocardium [30]. In addition, tyrosine phosphorylation facilitated by Src kinase was reported to promote calcium release from sarcoplasmic reticulum [31], which favors cellular calcium overload, which is also characteristic of AF [32].

Slowed impulse conduction is another mechanism related to Src that promotes atrial fibrillation. Propagation of the depolarization wave in the myocardium depends on the conductivity of gap junctions, which, in the atria, are formed mainly by connexin 43 (Cx43). Gap junction permeability relies primarily on phosphorylation of Cx43. Src, in addition to protein kinase C (PKC) and mitogen-activated protein kinases (MAPKs), directly phosphorylates Cx43, which leads to channel closure and the inhibition of gap junction-mediated intercellular communication [33]. It has also been shown that c-Src reduces the expression of Cx43 [34]. Moreover, other signaling enzymes that phosphorylate Cx43, like MAPKs and PKC, are themselves activated by Src [35,36]. Finally, c-Src kinase is involved in the disruption of Cx43 and ZO-1 coupling at the intercalated disc and the lateralization of connexins, which results in the slowing of longitudinal conduction of the depolarization wave [37,38].

Tyrosine kinases may also contribute to the modification of the anatomical substrate of AF. Enlargement of the atria and fibrosis of atrial tissue are the most common morphological alterations observed in AF. Extensive myocardial fibrosis of the myocardium has been described in mice with cardiac-specific overexpression of PDGF peptides [39,40]. Src kinase is activated downstream of PDGF receptors and mediates their cellular effects [41]. Increased expression of PDGF-B has been reported in patients with chronic AF, and both PDGF-B and collagen expression in canine atria rise with the increasing duration of AF [42]. Hemodynamic alterations, such as atrial pressure overload, which frequently accompany AF, induce infiltration of the myocardium by mast cells that release PDGF-A [43]. Fibroblasts may also serve as a source of PDGF [44]. Different PDGF isoforms stimulate fibroblast proliferation and collagen synthesis and may also affect the action potential of atrial cardiomyocytes, particularly those that maintain direct cellular contact with fibroblasts [44]. Our results show higher expression of PDGF receptors in patients with PoAF, which may facilitate PDGF signaling and thereby potentially enhance activation of Src, further promoting postoperative AF. We also observed that patients with PoAF had greater left atrial dimension and that this group exhibited higher c-Src expression. This suggests that Src may be linked not only to molecular signaling pathways but also to atrial enlargement and remodeling. c-Src appears to play a versatile role in this context; it may respond to mechanical stress and drive atrial enlargement and structural remodeling, while at the same time exerting a more specific arrhythmogenic influence [45]. This interplay between structural stress and Src signaling may underlie the development of a vulnerable atrial morphological and electrophysiological substrate for PoAF.

From a clinical perspective, postoperative atrial fibrillation remains a common complication despite the widespread use of AF substrate-modifying medications, such as beta blockers or angiotensin/aldosterone antagonists. Our preliminary results suggest that preoperative activation of c-Src kinase may represent a molecular substrate predisposing to PoAF. This finding is hypothesis-generating and requires confirmation in larger cohorts but points to two potential translational directions: c-Src activity as a biomarker of atrial vulnerability and Src signaling as a possible pharmacological target. Given that Src inhibitors are already available and further developed in other therapeutic areas, our data provide a rationale for further experimental and clinical studies to explore whether modulation of this pathway could contribute to the prevention of PoAF.

## 5. Limitations

The main limitation of this study is a relatively small sample size, which increases the risk of both type I and type II statistical errors and limits the generalizability of the findings. Given the observational nature of the study, the results should be considered hypothesis-generating, and potential causal relationships between the identified molecular alterations and the occurrence of PoAF should be interpreted with caution.

## 6. Conclusions

Our results suggest that elevated early c-Src activity may play a role in the development of postoperative atrial fibrillation. While further studies are needed to clarify its mechanistic contribution, the existence of Src-targeting drugs supports its potential as a therapeutic target. Notably, these findings broaden the scope of Src-related pathophysiology in atrial fibrillation, moving beyond its established role in chronic AF to implicate it in acute postoperative arrhythmia.

## Figures and Tables

**Table 1:** Basic characteristics of the examined groups.

	SR Group *N* = 28	PoAF Group *N* = 14	*p*
Age (years)	55.8 ± 15.5	61.8 ± 9.5	NS
Males (*n*; %)	18; 64%	8; 57%	NS
Concomitant disease:			
Diabetes mellitus (%)	17.9%	35.7%	NS
Hypertension (%)	46.5%	57.1%	NS
Indication for surgery (*n*):			
MVI	3	2	
MVS	0	0	
AI	0	2	
AS	4	3	
CAD	18	5	
Combined valvular	0	2	
Valvular + CABG	3	0	
Echocardiographic parameters:			
LA [mm]	38.9 ± 5.9	45.3 ± 6.5	*p* < 0.025
RA [mm]	36.6 ± 4.2	37.8 ± 3.8	NS
LVEDD [mm]	53 ± 8.4	53 ± 11	NS
RVEDD [mm]	25.1 ± 6.0	25.8 ± 3.0	NS
LVEF [%]	51.9 ± 12.3	45.5 ± 12.1	NS
Pharmacotherapy (*n*):			
ACE inhibitor/ARB	18	10	NS
Β-blocker	18	13	NS
Calcium channel blocker	4	4	NS
Statin	16	10	NS
Spironolactone	2	7	*p* < 0.05

Abbreviations: MVI—mitral valve insufficiency; MVS—mitral valve stenosis; AI—aortic valve insufficiency; AS—aortic valve stenosis; CAD—coronary artery disease; CABG—coronary artery bypass grafting; LA—left atrium; RA—right atrium; LVEDD—left ventricular end diastolic diameter; RVEDD—right ventricular end diastolic diameter; LVEF—left ventricular ejection fraction.

**Table 2:** Comparison of correlations between abundances of total and phosphorylated Src protein and key regulators of Src activity in both PoAF (left table) and SR (right table) groups. The table presents Spearman’s Rho values. Significant correlations (*p* < 0.001) are indicated by bold and italic font.

	SR Group		PoAF Group	
	c-Src	phospho-c-Src	c-Src	phospho-c-Src
STAT3	***0.5***	0.43	***0.93***	***0.9***
phospho-STAT3	***0.53***	***0.59***	***0.84***	***0.85***
ERK1/2	0.86	0.56	***0.87***	***0.9***
phospho-ERK1/2	***0.82***	0.59	***0.76***	0.75
PDGFRα	***0.69***	0.61	***0.81***	***0.87***
phospho-PDGFRα	0.84	0. 70	***0.84***	***0.95***
PDGFRβ	***0.71***	0.62	***0.85***	***0.88***
phospho-PDGFRβ	0.4	***0.75***	0.69	0.66

## Data Availability

Raw anonymized data is available from the corresponding author upon request.

## Abbreviations

The following abbreviations are used in this manuscript:

MDPI	Multidisciplinary Digital Publishing Institute
DOAJ	Directory of open access journals
AF	Atrial fibrillation
PoAF	Postoperative atrial fibrillation
SR	Sinus rhythm
ECG	Electrocardiogram
PDGF	Platelet-derived growth factor
PDGFR	Platelet-derived growth factor receptor
MAPK	Mitogen-activated protein kinase
